# Blockade of EP4 by ASP7657 Modulates Myeloid Cell Differentiation In Vivo and Enhances the Antitumor Effect of Radiotherapy

**DOI:** 10.1155/2023/7133726

**Published:** 2023-11-28

**Authors:** Toshihide Nishibata, Nobuaki Amino, Ruriko Tanaka-Kado, Susumu Tsujimoto, Tomoko Kawashima, Satoshi Konagai, Tomoyuki Suzuki, Masahiro Takeuchi

**Affiliations:** Immuno-oncology, Astellas Pharma Inc., 21 Miyukigaoka, Tsukuba, Ibaraki 305-8585, Japan

## Abstract

The tumor microenvironment (TME) is thought to influence the antitumor efficacy of immuno-oncology agents through various products of both tumor and stromal cells. One immune-suppressive factor is prostaglandin E_2_ (PGE_2_), a lipid mediator whose biosynthesis is regulated by ubiquitously expressed cyclooxygenase- (COX-) 1 and inducible COX-2. By activating its receptors, PGE_2_ induces immune suppression to modulate differentiation of myeloid cells into myeloid-derived suppressor cells (MDSCs) rather than dendritic cells (DCs). Pharmacological blockade of prostaglandin E receptor 4 (EP4) causes a decrease in MDSCs, reprogramming of macrophage polarization, and increase in tumor-infiltrated T cells, leading to enhancement of antitumor immunity in preclinical models. Here, we report the effects of the highly potent EP4 antagonist ASP7657 on the DC population in tumor and antitumor immune activation in an immunocompetent mouse tumor model. Oral administration of ASP7657 inhibited tumor growth, which was accompanied by an increase in intratumor DC and CD8^+^ T cell populations and a decrease in the M-MDSC population in a CT26 immunocompetent mouse model. The antitumor activity of ASP7657 was dependent on CD8^+^ T cells and enhanced when combined with an antiprogrammed cell death-1 (PD-1) antibody. Notably, ASP7657 also significantly enhanced the antitumor efficacy of radiotherapy in an anti-PD-1 antibody refractory model. These results indicate that the therapeutic potential of ASP7657 arises via upregulation of DCs and subsequent CD8^+^ T cell activation in addition to suppression of MDSCs in mouse models and that combining EP4 antagonists with radiotherapy or an anti-PD-1 antibody can improve antitumor efficacy.

## 1. Introduction

Surgery, radiotherapy, and chemotherapy have long been standards of care for cancer patients [1, 2]. Although complete local control before spread of a tumor from the primary site can be curative, surgical treatment and radiotherapy are thought to be ineffective in patients with advanced stage tumors or residual tumor mass after treatment. Further, although advances in combination chemotherapy and targeted therapy have significantly improved clinical outcomes for many tumor types, only a limited population of patients with advanced tumors achieves long-term complete response. Recent cancer therapy approvals have included immuno-oncology agents such as immune checkpoint inhibitors (ICI), which have durable clinical benefits for various tumor types [2]. ICIs stimulate T cell activity against tumor cells by blocking signaling pathways that inhibit this function, leading to long-lasting antitumor effects and even tumor-free cases [2, 3]. However, since the response rate to these drastic antitumor mechanisms remains limited, researchers are currently investigating a number of challenges to improve the clinical benefits of immuno-oncology agents [4].

Given that adaptive immunity against tumor cells is mainly dependent on tumor antigen-specific T lymphocytes [5], antigen presentation by dendritic cells (DCs) that leads to priming and activation of effector T cells is crucial to the effective induction of antitumor immunity [6]. In this context, DC recruitment to the tumor site is also considered a promising strategy through which to induce antitumor immunity, since tumor-infiltrated DCs capture and present tumor-specific antigens to activate tumor-recognizing T cells [7]. Another approach by which to harness the antitumor activity of cytotoxic T lymphocytes (CTLs) is to ameliorate the immune-suppressive tumor microenvironment (TME) such as by decreasing the population of infiltrated myeloid-derived suppressor cells (MDSCs), regulatory T cells (Tregs), and tumor-associated macrophages (TAMs). Strategies to deplete TAMs [8] or Tregs [9] are also being tested in the clinic. Thus, ways to reduce the numbers of these immune-suppressing cells from tumors are also attracting attention.

Prostaglandin E_2_ (PGE_2_) is a lipid mediator whose biosynthesis is regulated by ubiquitously expressed cyclooxygenase- (COX-) 1 and inducible COX-2. Although distributed ubiquitously, it is more abundant in colon tumors than normal mucosa [10]. It exerts its various biological effects by binding receptors EP1-EP4 and is involved in tumor-related immune suppression. In vitro studies suggest that PGE_2_ plays a role in the differentiation and maturation of myeloid cells including suppression of DCs and induction of MDSCs and TAMs. In the presence of PGE_2_, human CD14^+^ monocytes differentiate into Th2-inducing DCs, or even into monocytic MDSCs (M-MDSCs), while differentiating into Th1-inducing CD1a^+^ DCs in the absence of PGE_2_ [11–13]. PGE_2_ also inhibits the activation of immune-stimulatory M1-TAMs [14] and reportedly induces polarization of M1-TAMs to M2-TAMs [15]. In addition, PGE_2_ promotes induction and activation of Tregs through EP4 receptors in a mouse model [16] through the production of CCL17 and CCL22 from recently identified mregDCs (mature DCs enriched in immunoregulatory molecules) in response to PGE_2_-EP2/EP4 signaling pathways [17]. Consistently, EP4 antagonists reverse suppression of the differentiation of monocytes into DCs in vitro [18] and in vivo [19], inhibit protumor myeloid cell differentiation, and inhibit polarization of mouse peritoneal macrophages into an M2-like phenotype [20]. In addition, while EP4 antagonists also show antitumor effects in monotherapy, combining them with an anti-PD-1 antibody and Treg-targeted interleukin (IL)-2-diphtheria toxin fusion protein has been shown to enhance their antitumor effects [18, 19, 21, 22]. Thus, the PGE_2_-EP4 axis, which regulates multiple immune suppression mechanisms, is expected to be a promising target for novel immune-oncology agents as a result of their switching the fate of myeloid cells from immune-suppressive MDSCs or M2-TAMs to immune-stimulating DCs or M1-TAMs and consequent amelioration of immune-suppressive TMEs.

Several EP4 antagonists are currently being tested in clinical trials. Here, we investigated the effects of ASP7657, a highly potent and selective EP4 antagonist [23], on myeloid cells including DCs in vitro and in vivo, and tumor growth inhibition through activation of antitumor immunity. We observed a significant improvement in the antitumor response of ASP7657 when combined with radiotherapy in an anti-PD-1 refractory model, suggesting that EP4 antagonists can be potentially used in combination with not only ICIs but also radiotherapy in the clinic.

## 2. Results

### 2.1. Effects of ASP7657 on Myeloid Cell Differentiation

**Figure 1:**
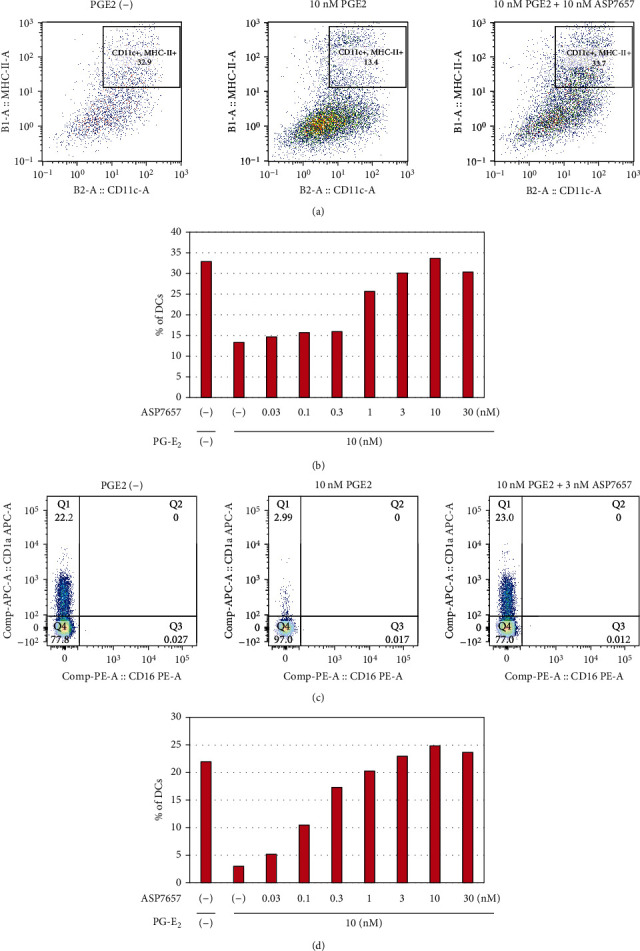
Effect of ASP7657 on PGE_2_-mediated suppression of the differentiation of mouse BM cells and hCD14^+^ monocytes into DCs in vitro: (a, c) representative flow cytometry dot plots; (b, d) effect of ASP7657 on the DC population; (a, b) mouse BM cells were cultured with 10 ng/mL mGM-CSF in the presence or absence of 10 nM PGE_2_ for 7 days; (c, d) human CD14^+^ monocytes were cultured with 50 ng/mL hGM-CSF and 50 ng/mL hIL-4 in the presence or absence of 10 nM PGE_2_ for 7 days; (a, c) PGE_2_ suppressed differentiation of mouse BM cells and human CD14^+^ monocytes into DCs in vitro; (b, d) addition of ASP7657 throughout the culture period reversed PGE_2_-mediated suppression of DC differentiation at the indicated doses. Shown are representative results of 2 independent assays.

PGE_2_ reportedly regulates the differentiation of myeloid cells into DCs and MDSCs in vitro [11]. Thus, we examined whether ASP7657 reverses PGE_2_-mediated suppression of the differentiation of mouse BM cells (Figures 1(a) and 1(b)) and human CD14^+^ monocytes isolated from peripheral blood mononuclear cells (PBMCs) (Figures 1(c) and 1(d)) into DCs in vitro. In mouse BM cell culture, 10 nM PGE_2_ reduced the population of CD11c^+^ major histocompatibility complex (MHC) class II^+^-activated DCs induced by mGM-CSF at 10 ng/mL (Figure 1(a)). ASP7657 reversed the PGE_2_-driven suppression of the differentiation of monocyte precursors into DCs (Figure 1(b)). Similarly, 10 nM PGE_2_ also inhibited differentiation of human CD14^+^ human monocytes into CD1a^+^ CD16^−^ cells induced by hGM-CSF (50 ng/mL) and hIL-4 (50 ng/mL) (Figure 1(c)), and ASP7657 reversed this suppression at a slightly lower concentration than that noted in the mouse BM cell experiments (Figure 1(d)). These results demonstrate that ASP7657 can reverse PGE_2_-driven suppression of the differentiation of CD14^+^ monocytes into DCs in a similar manner to other EP4 antagonists in vitro.

**Figure 2:**
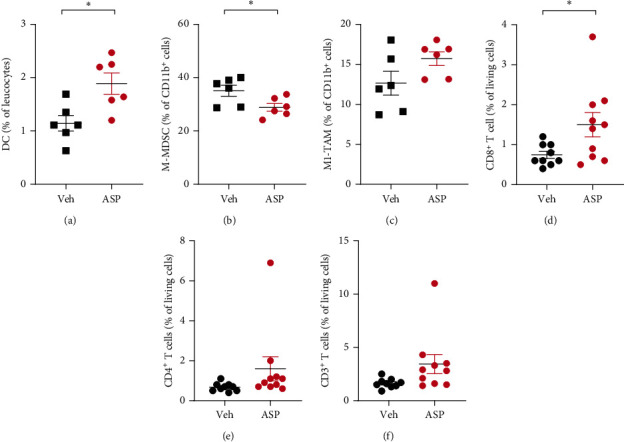
Analysis of tumor-infiltrating immune cells in CT26.WT tumors. CT26.WT cells were inoculated (1 × 10^6^ (a–c) or 6 × 10^5^ (d–f) cells per mouse) into the right flank of BALB/c mice. After tumors were established, mice were treated with ASP7657 twice per day. (a–c) Modification of the myeloid cell population. Tumors were excised from mice treated for 10 days, enzymatically dissociated, stained, and analyzed using flow cytometry (*n* = 6 per group). Frequencies of DCs (a, CD45^+^ CD11b^+^ CD11c^+^ MHC-II^high^ 7-AAD^−^ cells in CD45^+^ 7-AAD^−^ cells), M-MDSCs (b, CD45^+^ CD11b^+^ Ly6G^low^ Ly6C^high^ 7-AAD^−^ cells in CD45^+^ CD11b^+^ 7-AAD^−^ cells), and M1-TAMs (c, CD45^+^ CD11b^+^ F4/80^+^ MHC-II^high^ 7-AAD^−^ cells in CD45^+^ CD11b^+^ 7-AAD^−^ cells) are plotted. Modification of the T cell population. Tumors were excised from mice treated for 14 days, enzymatically dissociated, stained, and analyzed using flow cytometry (*n* = 9 − 10 per group). Frequencies of CD8^+^ T cells (d, CD45^+^ CD3^+^ CD4^−^ CD8^+^ 7-AAD^−^ cells), CD4^+^ T cells (e, CD45^+^ CD3^+^ CD4^+^ CD8^−^ 7-AAD^−^ cells), and conventional T cells (f, CD45^+^ CD3^+^ 7-AAD^−^ cells) in 7-AAD^−^ cells are plotted. Veh: vehicle; ASP: ASP7657 3 mg/kg/day. Data show mean ± SEM. ^∗^*p* < 0.05 compared to the vehicle-treated group (Student's *t*-test).

To investigate the effect of ASP7657 on the population of myeloid cells in tumors, we established a CT26.WT immune-competent syngeneic mouse tumor model and used flow cytometry to analyze the immune cell population after twice daily administration of ASP7657 at 3 mg/kg/day for 10 days. Tumors were excised and dissociated enzymatically to prepare single-cell suspensions and stained and analyzed using a flow cytometer. At day 10, mean intratumor DC (CD45^+^ CD11b^+^ CD11c^+^ MHC class II^high^) populations in the control and ASP7657-treated groups were 1.14% and 1.89%, respectively (Figure 2(a)). Mean M-MDSC (CD45^+^ CD11b^+^ Ly6C^high^, Figure 2(b)) and M1-like macrophage (CD45^+^ CD11b^+^ F4/80^+^ MHC class II^high^, Figure 2(c)) populations in the control and ASP7657-treated groups were 35.1% and 28.9% and 12.6% and 15.7%, respectively. Treatment with ASP7657 for 10 days significantly increased the DC and decreased the M-MDSC population in CT26.WT tumors, suggesting that ASP7657 can ameliorate the TME by facilitating differentiation of immune-activating myeloid cells.

### 2.2. Effects of ASP7657 on T Cell-Mediated Antitumor Immunity

**Figure 3:**
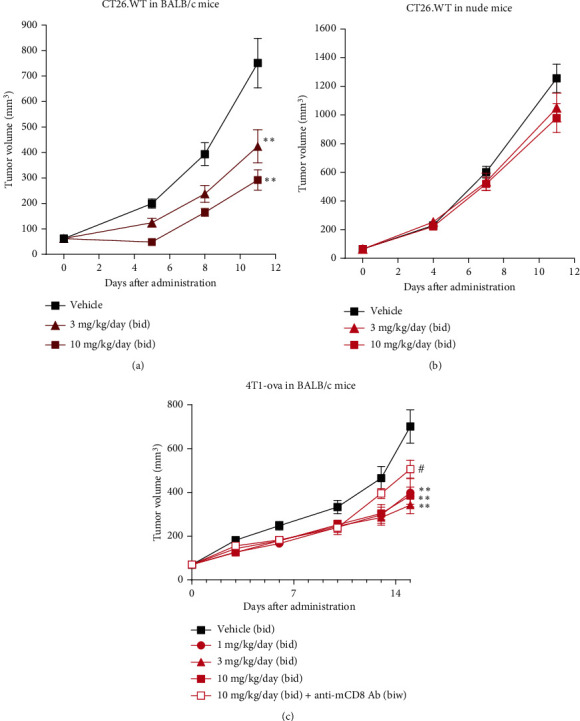
ASP7657 activates T cell-mediated antitumor activity. (a, b) CT26.WT cells were inoculated (1 × 10^6^ (a) or 6 × 10^5^ (b) cells per mouse) into the right flank of BALB/c (a) and nude mice (b). After tumors were established, mice were treated with ASP7657 twice per day. (c) 4T1-ova cells were inoculated (1.5 × 10^4^ cells per mouse) into the right mammary fat pad of BALB/c mice. After tumors were established, mice were treated with ASP7657 twice per day and injected with an anti-mCD8 antibody at 0.2 mg/head twice per week (biw). Data show mean ± SEM for *n* = 9 − 10 mice per group. ^∗∗^*p* < 0.01, compared with the vehicle group (Dunnett's multiple comparison test); ^#^*p* < 0.05, compared with the 10 mg/kg/day group (Student's *t*-test).

To evaluate the effects of ASP7657 on antitumor immunity in vivo, we analyzed the T cell populations in CT26.WT tumors in a similar manner to that described above. A significant increase in the CD8^+^ T cell population was observed on day 14 (Figure 2(d)). Mean CD8^+^ T cell populations in the control and ASP7657-treated groups were 0.74% and 1.5%, respectively. Although not significant, increases in the CD4^+^ T cell (Figure 2(e)) and pan T cell (Figure 2(f)) populations were also observed. Mean CD4^+^ T cell and pan T cell populations in the control and ASP7657-treated groups were 0.67% and 1.6% and 1.6% and 3.5%, respectively. Next, we examined whether the antitumor effects of ASP7657 were demonstrable in immune-competent settings and abolished in the absence of functional T cells in an immune-compromised nude mouse model and CD8^+^ T cell depletion model. In a CT26.WT subcutaneous transplantation model using immune-competent BALB/c mice, ASP7657 significantly inhibited tumor growth at 3 and 10 mg/kg/day (Figure 3(a)). In contrast, ASP7657 did not inhibit tumor growth when administered to immune-deficient mice (Figure 3(b)). While ASP7657 significantly inhibited tumor growth in a 4T1-ova orthotopic transplantation (OT) model, this effect was impaired when the drug was combined with an anti-mCD8 antibody (Figure 3(c)). Together, these findings demonstrate that ASP7657 exerts its antitumor efficacy via CD8^+^ T cell-dependent antitumor immunity.

### 2.3. ASP7657 Shows Antitumor Efficacy in Multiple Syngeneic Mouse Tumor Models

**Figure 4:**
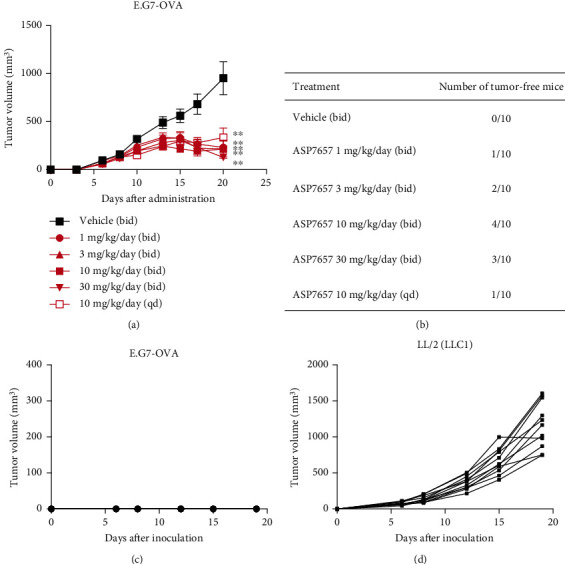
Rechallenged CR mice show no tumor growth. (a) Antitumor activity of ASP7657 in a syngeneic mouse E.G7-OVA-engrafted model. On day 0, E.G7-OVA cells were inoculated (5 × 10^5^ cells per mouse) into the right flank of C57BL/6J mice, and oral administration of ASP7657 was started once per day (qd) or twice per day (bid). Significant inhibition of tumor growth was seen in all treatment groups at day 20. Data show mean ± SEM for *n* = 10 mice per group. ^∗∗^*p* < 0.01, compared with the vehicle-treated group (Dunnett's multiple comparison test). (b) Number of mice in (a) that achieved CR. E.G7-OVA tumors had regressed completely in 11 out of 50 ASP7657-treated mice by day 34, and these animals remained tumor-free for an additional 8 weeks. (c, d) The 11 CR mice from (b) were reinoculated with E.G7-OVA cells (c) into the right flank and with LL/2(LLC1) cells (d) into the left flank simultaneously at 5 × 10^5^ cells per mouse. Mice were observed for 19 days. No tumor growth was detected in the right flank whereas aggressive tumor growth was seen in the left flank.

In addition to CT26.WT and 4T1-ova OT models, we also evaluated the antitumor activity of ASP7657 in other tumor models. Administration of ASP7657 significantly inhibited tumor growth in the E.G7-OVA (Figure 4(a)), EMT-6 (Figure S2A), and 4T1 (Figure S2B) models. A tendency toward growth inhibition was also shown in the LL/2(LLC1) (Figure S2C) model. In contrast, no growth inhibition effect was observed in the B16-F10 model (Figure S2D). These results suggest that ASP7657 may be effective against multiple tumor types. Of note, oral administration of ASP7657 at all indicated doses rendered 11 of 50 treated mice tumor-free (Figure 4(b)). These tumor-free mice were monitored for 10 weeks without further treatment to confirm complete regression (CR).

### 2.4. Rechallenging CR Mice with E.G7-OVA Reveals Antigen-Specific Immunological Memory

To investigate the establishment of immunological memory, we reinoculated CR mice with E.G7-OVA. After 10 weeks from discontinuation of ASP7657 treatment, CR mice were inoculated with E.G7-OVA in the right flank and LL/2(LLC1) in the left frank simultaneously. While E.G7-OVA did not grow in any CR mice (Figure 4(c)), LL/2(LLC1) showed normal growth (Figure 4(d)). This result suggests that CR mice treated with ASP7657 developed tumor antigen-specific immunological memory that was maintained for more than 2 months.

### 2.5. Effect of ASP7657 in Combination with Anti-PD-1 Antibody or Radiotherapy

**Figure 5:**
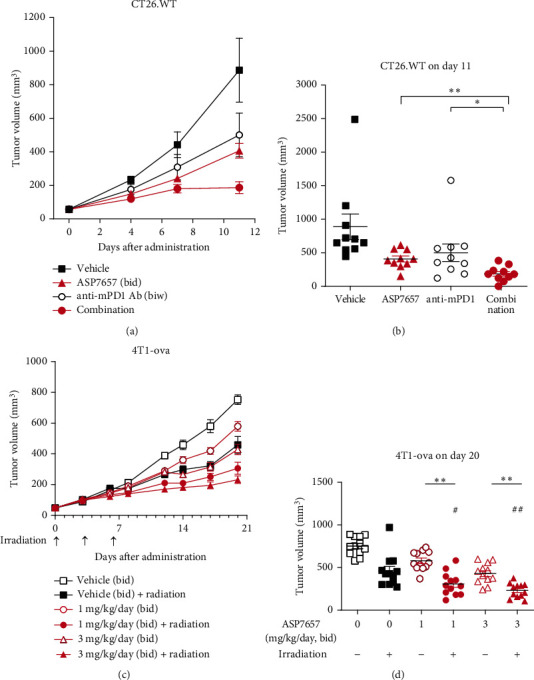
Effect of combining ASP7657 with anti-PD-1 antibody in CT26.WT or radiotherapy in the 4T1-ova model. (a, b) CT26.WT cells were inoculated (1 × 10^6^ cells per mouse) into the right flank of BALB/c mice (*n* = 10 per group). After tumors were established, mice were treated with ASP7657 twice per day at 3 mg/kg/day and with an anti-mPD-1 antibody or isotype control at 0.1 mg/head twice per week. Data show mean ± SEM. ^∗^*p* < 0.05, ^∗∗^*p* < 0.01, compared with each single agent group (Student's *t*-test). (c, d) 4T1-ova cells were inoculated (1.5 × 10^4^ cells per mouse) into the right mammary fat pad of BALB/c mice (*n* = 12 per group). After tumors were established, mice were treated with ASP7657 twice per day at 1 or 3 mg/kg/day and irradiated with 5 Gy X-rays on days 0, 3, and 6. Data show mean ± SEM. ^∗∗^*p* < 0.01, compared with the ASP7657+sham radiation group (Student's *t*-test); ^#^*p* < 0.05, ^##^*p* < 0.01, compared with the vehicle+radiation group (Dunnett's multiple comparison test).

Since blocking the PD-1/PD-L1 axis potentiates CD8^+^ T cell-dependent antitumor immunity [2], we hypothesized that combining ASP7657 with an anti-PD-1 antibody would enhance antitumor efficacy. To confirm this, we evaluated the combined antitumor effect of ASP7657 with an anti-mPD-1 antibody in the CT26 tumor-engrafted mouse model (Figures 5(a) and 5(b)). At day 11, ASP7657 and the anti-mPD-1 antibody alone inhibited tumor growth by 58% and 47%, respectively. Combination treatment significantly increased the tumor growth inhibition rate to 84%. In addition, complete tumor regression was observed in 2 of 10 mice in the combination treatment group at day 21 (data not shown). This result suggests that combining ASP7657 with an anti-PD-1 antibody may enhance antitumor immunity.

Radiotherapy is also widely used to treat cancer. Since the effect of single high-dose radiotherapy depends on activation of DCs and an increase in tumor-specific effector CD8^+^ T cells [24], we speculated that combining radiotherapy with an EP4 antagonist would likely enhance treatment efficacy. Therefore, we explored the combination of ASP7657 with radiotherapy in the 4T1-ova OT model (Figures 5(c) and 5(d)). As expected, the combination of ASP7657 and radiation significantly enhanced inhibition of tumor growth compared to each treatment alone. These results suggest that radiotherapy can also be used in combination with ASP7657.

## 3. Discussion

Rather than directly targeting effector cells, recent approaches aimed at manipulating immune-suppressive myeloid cells or their products have attracted attention as a strategy to restore patients' intrinsic antitumor immunity. Several of these are under clinical investigation, including compounds that inhibit arginase or indoleamine 2,3-dioxygenase (IDO) [25, 26], and suppress TAM infiltration by blocking colony-stimulating factor 1 receptor (CSF-1R) [8]. Polarization of M2-TAMs into M1-TAMs is a second approach to stimulating antitumor immunity [10]. However, given that these monotherapies do not demonstrate clear antitumor effects in the clinic, targeting individual factors may be insufficient to release immune suppression, probably due to the existence of multiple mechanisms. PGE_2_ is a multifunctional lipid mediator whose signal is transmitted via four receptors to it. PGE_2_ regulates multiple immune suppression mechanisms, including the suppression of DCs and induction of MDSCs, TAMs, and Tregs. In addition to this effect on immunosuppressive cells, PGE_2_-EP4 signaling directly inhibits natural killer (NK) cell activity [27] and PGE_2_ suppresses IFN-*γ* expression in CD8^+^ T cells [28]. As cross-talk between myeloid cells and effectors, it is also reported that the addition of a PGE_2_ or EP4 agonist inhibits IL-12 and TNF-*α* production by monocyte-derived DCs [29]. Since EP4 has higher affinity for PGE_2_ than prostaglandin E receptor 2 (EP2) [30], EP4 antagonists may be more effective for blocking PGE_2_ signaling in certain settings even though both EP2 and EP4 are coupled to stimulatory G-proteins and lead to downstream cAMP elevation. ASP7657 is an orally available, highly potent and selective EP4 antagonist with a Ki value of 2.21 nM [23]. In this study, we demonstrated that ASP7657 reversed PGE_2_-dependent inhibition of the differentiation of mouse BM cells and human CD14^+^ monocytes into DCs in vitro. Since M-MDSCs were not efficiently induced in this culture system, future studies should evaluate the inhibitory effect of ASP7657 on M-MDSC induction from mouse BM cells and human CD14^+^ monocytes as well as the induction and polarization of macrophages. Consistent with these in vitro results, administration of ASP7657 increased the activated DC population in tumors in vivo. In addition, a decrease in the M-MDSC population and nonsignificant trend toward an increase in M1-TAMs were also observed. Since the TAM population is in a state of constant transition between the M1 and M2 types, further clarification of macrophage subpopulation using additional markers such as iNOS, CXCR1, and CD206 appears necessary. Collectively, these results indicate that ASP7657 has the potential to alleviate immune evasion caused by PGE_2_ in the TME. In the future, comprehensive analysis of the effect of ASP7657 on population change of myeloid cells in tumors, including detailed classification of DC subsets such as cDC1 and cDC2, would help us to more precisely understand the effect of EP4 signaling on the CD8^+^ T cell activation process.

Consistent with the previous reports [18, 19, 21], treatment with ASP7657 significantly increased the population of CD8^+^ T cells and showed a trend towards increasing CD4^+^ and total T cells in tumors. In line with other EP4 antagonists, ASP7657 also demonstrated antitumor effects in immune-competent syngeneic mouse tumor models when used as a monotherapy. Notably, ASP7657 alone caused CR of E.G7-OVA tumors. Rejection of reinoculated E.G7-OVA tumor cells suggested that ASP7657 treatment established immunological memory that was maintained for at least 2 months after CR. In addition, we demonstrated that the antitumor effect of ASP7657 was dependent on T cell-mediated immunity through studies using immune-deficient nude mice and depletion of CD8^+^ T cells in immune-competent mice. Given that the antitumor effect of the combined blockade of EP2 and EP4 in the ICI-insensitive LLC1 model is independent of CD8^+^ T cells [27], the contribution of CD8^+^ T cells is likely affected by the immunological tumor microenvironment, including the tumor's immunogenicity and/or intrinsic sensitivity to ICIs. Another mechanism of PGE_2_-mediated immune suppression is the enhancement of Treg function, as evidenced by the finding that combination treatment comprising an EP4 plus an EP2 antagonist reduced the number of tumor-infiltrating Tregs in vivo and PGE_2_-mediated stabilization of Tregs in vitro [17]. One of the mechanisms of immunosuppression by Tregs is inhibition of the priming of naïve T cells through CTLA-4-mediated blockade of the costimulatory CD80/CD86 signaling which accompanies antigen presentation by activated DCs [9]. Thus, ASP7657 could increase primed and activated effector T cells via both Treg-dependent and Treg-independent mechanisms considering the increase in activated DCs in tumors. Although nude mice lack both Tregs and functional CD8^+^ T cells, our depletion study illustrated involvement of CD8^+^ T cells in the antitumor effect of ASP7657. Taken together, these findings suggest that ASP7657 modulates the myeloid cell population including increasing DCs in tumors, leading to the activation of T cell-mediated antitumor immunity and the subsequent inhibition of tumor growth or even CR in certain settings. This notion is supported by the fact that the antitumor effects of ASP7657 significantly improved when it was combined with an anti-PD-1 antibody, which enhances T cell-mediated tumor cell killing through blockade of coinhibitory PD-1 receptors expressed on activated T cells, potentially increased as a result of ASP7657 treatment. Further research on the activation status of tumor-infiltrating DCs and changes in subpopulations of DCs such as CD8^+^ T cell-inducing CD103^+^ DCs will help identify the immune activation mechanisms induced by ASP7657. These studies will likely suggest the potential of combining ASP7657 with DC activators, which upregulate the expression of costimulatory molecules. In terms of the mechanism by which ASP7657 ameliorates the immune-suppressive TME, we hypothesize that multiple factors may be involved. MDSCs reportedly inhibit antitumor immunity by producing MDSC-associated factors such as arginase, reactive oxygen species, IL-10, IDO-1/2, and PGE_2_ [31]. M2-TAMs support tumor growth by producing ornithine, VEGF, EGF, TGF-*β*, and PGE_2_ [14]. Tregs also secrete TGF-*β* and IL-10, directly interact with cells, and consume the proimmune cytokine IL-2 [32]. These factors collectively paralyze antitumor effector cells by suppressing activation, proliferation, and survival. Indeed, a lower M-MDSC frequency in peripheral blood is correlated with favorable clinical outcomes in ipilimumab-treated melanoma patients [33]. Measurement of immune-suppressive products such as cytokines, chemokines, and PGE_2_ will help improve understanding of other mechanisms by which ASP7657 activates antitumor immunity.

A variety of conventional chemotherapies induce immunogenic cell death (ICD) in tumor cells, which is characterized by translocation of calreticulin to the cell surface and release of damage-associated molecular pattern (DAMP) molecules to recruit and activate antigen-presenting cells including DCs and M1-TAMs [34]. Radiotherapy is one treatment for tumor cells that induces ICD [35]. Historically, a pivotal mechanism of action of radiotherapy was thought to be direct induction of tumor cell death. However, the importance of immune activation in inducing antitumor efficacy was reported in a preclinical study which demonstrated that the antitumor effect of single high-dose radiation crucially depends on DCs and CD8^+^ T cells [24] and also in a systematic review which showed the clinical benefits of the combination of radiotherapy with ICIs in non-small-cell lung cancer [36]. Given that ASP7657 increased the DC population in tumors, we hypothesized that combining it with treatments which cause ICD could be beneficial. In this work, we demonstrated that ASP7657 significantly enhanced the antitumor effects of radiotherapy in a syngeneic 4T1-ova orthotopic transplantation model, as expected. In humans, there have been reports of frequent overexpression of COX-2 in many tumor types [10, 37–42] and increased COX-2 expression and PGE_2_ production by tumor cells in response to genotoxic stress induced by radiation or doxorubicin treatment [43]. In addition, involvement of the PGE_2_-EP4 axis in ultraviolet exposure-induced systemic immunosuppression has also been reported [44]. Therefore, amelioration of the immune-suppressive TME may also contribute a positive effect on combination treatment with radiotherapy by targeting the PGE_2_ signaling pathway. Further investigation of population changes in CD8^+^ and CD4^+^ T cells along with myeloid cells, especially cDC1 subsets, will lead to better understanding of the mechanisms by which the combination of ASP7657 with radiation activates the immune response, including the enhancement of antigen cross-presentation.

In conclusion, we showed that ASP7657 is a potent and orally active antitumor agent that modifies the myeloid population in tumors and subsequent activation of antitumor immunity mediated by CD8^+^ T cells by blocking PGE_2_-mediated immune suppression. The potential of combination therapy with ICIs and radiotherapy was also demonstrated. These findings are mostly consistent with recent reports on other EP4 antagonists [18, 19, 21, 22] except for the demonstration of a positive effect when combined with radiotherapy. Our work supports evidence of the clinical potential of EP4 antagonists as monotherapy and in combination with ICIs or radiotherapy. Since various standard treatment regimens exist, further evaluation is needed to clarify which combinations are effective for which tumor types. The possibility that triple combinations comprising EP4 antagonists, ICIs, and radiotherapy or chemotherapy which induce ICD of tumor cells might be more beneficial should also be explored. Although several EP4 antagonists are currently being tested in clinical trials in combination with ICIs and chemoradiotherapy, further studies are needed to identify a predictive biomarker of response in patients and the best combination partner to maximize antitumor potential.

## 4. Materials and Methods

### 4.1. Reagents

ASP7657 was synthesized at Astellas Pharma Inc. (Tokyo, Japan). ASP7657 was dissolved in dimethyl sulfoxide or suspended in 0.5% methyl cellulose for in vitro and in vivo experiments, respectively. Recombinant mouse and human GM-CSF (mGM-CSF and hGM-CSF, respectively) were purchased from PeproTech Inc. (Rocky Hill, NJ, USA), and recombinant human IL-4 (hIL-4) was purchased from R&D Systems Inc. (Minneapolis, MN, USA). ACK Lysing Buffer, monothioglycerol, and Accutase™ were purchased from Thermo Fisher Scientific Inc. (Waltham, Massachusetts, USA), FUJIFILM Wako Pure Chemical Corporation (Osaka, Japan), and M&S Techno Systems Inc. (Osaka, Japan), respectively. Lymphocyte growth medium (LGM)-3 was purchased from Lonza Japan (Tokyo, Japan). PGE_2_ was purchased from Cayman Chemical (Ann Arbor, Michigan, USA). Anti-mCD8 (clone 53-6.7, catalog #BP0004-1) and anti-mPD-1 (clone RMP1-14, catalog #BE0146) antibodies were purchased from Bio X Cell (West Lebanon, NH, USA).

### 4.2. Cell Culture

E.G7-OVA, CT26.WT, and LL/2(LLC1) were purchased from ATCC (Manassas, VA, USA). 4T1-ova was established by Astellas Pharma Inc. E.G7-OVA and LL/2(LLC1) were cultured with DMEM supplemented with 10% fetal bovine serum (FBS). CT26.WT was cultured with RPMI1640 or DMEM containing 10% FBS. 4T1 was cultured with RPMI1640 containing 10% FBS. 4T1-ova was cultured with RPMI1640 supplemented with 10% FBS and 0.3 mg/mL Zeocin (Thermo Fisher Scientific Inc.). EMT-6 was cultured with Waymouth's medium supplemented with 15% FBS. Poietics CD14^+^ monocytes purchased from Lonza (Tokyo, Japan) were cultured with LGM-3.

### 4.3. Animals

All mice were purchased from Charles River Laboratories Japan Inc. (Kanagawa, Japan). Female BALB/c mice aged 8 weeks were used to obtain bone marrow (BM) cells. For studies examining the in vivo antitumor activity of ASP7657, female C57BL/6J and BALB/c mice aged 5-9 weeks were used. Animal studies were approved by the Institutional Animal Care and Use Committee of Astellas Pharma Inc., Tsukuba Research Center, which is accredited by the Association for Assessment and Accreditation of Laboratory Animal Care (AAALAC) International.

### 4.4. In Vitro Differentiation

BM cells were flushed from the femur and tibia of BALB/c mice with sterile culture media. Single-cell suspensions were cultured in RPMI1640 medium containing 10% FBS, 10 ng/mL mGM-CSF, and 0.5 mM monothioglycerol for 7 days in the presence of PGE_2_ (10 nM) and ASP7657 at indicated concentrations, with fresh medium added twice during the culture period. Human CD14^+^ monocytes were cultured in LGM-3 supplemented with 50 ng/mL hGM-CSF and 50 ng/mL hIL-4 for 7 days in the presence of PGE_2_ (10 nM) and ASP7657 at indicated concentrations with fresh medium added once during the culture period.

### 4.5. In Vivo Studies

CT26.WT cells were inoculated subcutaneously into the flank of female BALB/c and nude mice (BALB/c nu/nu) at 6 × 10^5^ or 1 × 10^6^ cells/0.1 mL/mouse. 4T1-ova cells were inoculated into the mammary fat pad of female BALB/c mice at 1.5 × 10^4^ cells/0.02 mL/mouse. EMT-6 and 4T1 cells were transplanted subcutaneously into the flank of female BALB/c mice at 1 × 10^5^ and 5 × 10^5^ cells/0.1 mL/mouse, respectively. E.G7-OVA, LL/2(LLC1), and B16-F10 cells were inoculated subcutaneously into the flank of female C57BL/6J mice at 5 × 10^5^, 3 × 10^5^ or 5 × 10^5^, and 3 × 10^5^ cells/0.1 mL/mouse, respectively. ASP7657 (0.1-30 mg/kg/day) was orally administered once or twice daily as indicated. Anti-mCD8 or anti-mPD-1 antibodies were intraperitoneally administered twice weekly at 0.2 or 0.1 mg/mouse, respectively. In combined treatment with radiotherapy, tumors were irradiated with X-rays (5 Gy) using MBR-1520R (Hitachi Power Solutions Co. Ltd.) on days 0, 3, and 6. Tumor volume was calculated as length × width^2^ × 0.5. Percent inhibition of tumor growth (% Inh) was calculated using the following formula: %Inh = 100 × (1 − [mean tumor volume on the day of measurement − mean tumor volume on the day of treatment initiation] in each group ÷ [mean tumor volume on the day of measurement − mean tumor volume on the day of treatment initiation] in the control group).

### 4.6. Flow Cytometry

Tumor Dissociation Kit, Mouse was purchased from Miltenyi Biotec GmbH (Bergisch Gladbach, Germany). The following antibodies were purchased from BD Biosciences (San Jose, CA, USA): anti-mCD16/32 (catalog #553142), Human BD Fc Block (catalog #564220), anti-mCD11b-APC-Cy7 (catalog #561039), anti-Ly6C-PE-Cy7 (catalog #560593), anti-Ly6G-FITC (catalog #551460), anti-mCD3e-APC-Cy7 (catalog #557596), anti-mouse I-A/I-E-FITC (catalog #553623), anti-mCD11c-APC (catalog #550261), anti-mCD11c-PE (catalog #557401), anti-hCD16-PE (catalog #555407), anti-hCD1a-APC (catalog #559775), and 7-amino-actinomycin D (7-AAD, catalog #559925). The following antibodies were purchased from BioLegend Inc. (San Diego, CA, USA): anti-mCD45-Pacific Blue (catalog #103126), anti-mCD45-Brilliant Violet 510 (catalog #103138), and anti-mCD4-PE (catalog #100408). Anti-mCD8a-FITC (catalog #11-0081-82) and anti-mF4/80-APC (catalog #11-4801-82) were purchased from Thermo Fisher Scientific Inc. To prepare single-cell suspensions of tumors, excised tumors were minced and digested with the Tumor Dissociation Kit, Mouse according to the manufacturer's instructions. Single-cell suspensions of cultured BM cells, CD14^+^ human monocytes, and dissociated tumor cells were blocked with anti-mCD16/32 or Human BD Fc Block, stained with fluorescently labeled antibodies by incubating on ice for 20 min, washed three times, and stained with 7-AAD solution before being analyzed on a BD FACSVerse (BD Biosciences) or MACSQuant Analyzer 10 (Miltenyi Biotec).

### 4.7. Statistical Analysis

Values are expressed as mean ± SEM. Differences between groups were analyzed using Dunnett's multiple comparison test or Student's *t*-test. All data analysis was performed using SAS statistical software (SAS Institute, Cary, NC, USA) or GraphPad Prism (version 8), with *p* values < 0.05 considered significant.

## Data Availability

The data used to support the findings of this study are available from the corresponding author upon reasonable request.

## Abbreviations

TME: Tumor microenvironment
PGE_2_: Prostaglandin E_2_
COX: Cyclooxygenase
MDSC: Myeloid-derived suppressor cell
DC: Dendritic cell
EP4: Prostaglandin E receptor 4
PD-1: Programmed cell death-1
ICI: Immune checkpoint inhibitor
CTL: Cytotoxic T lymphocyte
Treg: Regulatory T cell
TAM: Tumor-associated macrophage
GM-CSF: Granulocyte-macrophage colony-stimulating factor
7-AAD: 7-Amino-actinomycin D
PBMC: Peripheral blood mononuclear cell
OT: Orthotopic transplantation
CR: Complete regression
biw: Twice per week
qd: Once per day
bid: Twice per day
CSF-1R: Colony-stimulating factor 1 receptor
NK: Natural killer
IDO: Indoleamine 2,3-dioxygenase
EP2: Prostaglandin E receptor 2
ICD: Immunogenic cell death
DAMP: Damage-associated molecular pattern.

## Supplementary Materials

Supplementary Materials The supplementary information Figure S 1A-1B and Figure S 2A-2D are available in the supplementary material document. Figure S1: gating strategy for Figures 1(a) and 1(a). Figure S2: antitumor effect of ASP7657 in various tumors: (A–D) EMT-6 (1 × 10^5^ cells per mouse, A) and 4T1 (5 × 10^5^ cells per mouse, B) were subcutaneously inoculated into the right flank of BALB/c mice and LL/2(LLC1) (3 × 10^5^ cells per mouse, C) and B16-F10 (3 × 10^5^ cells per mouse, D) into the right flank of C57BL/6J mice. After tumors were established, mice were treated with ASP7657 twice per day at the indicated doses. Data show mean ± SEM (*n* = 9 − 10 per group). ^∗^*p* < 0.05, ^∗∗^*p* < 0.01, compared with the vehicle-treated group (Dunnett's multiple comparison test, except for LL/2(LLC1) (Student's *t*-test)).
