# Supplementary figures and images for: Blockade of EP4 by ASP7657 Modulates Myeloid Cell Differentiation In Vivo and Enhances the Antitumor Effect of Radiotherapy
Source: Biomed Res Int. 2023 Nov 28;2023:7133726. doi: 10.1155/2023/7133726 (PMC10697779; doi:10.1155/2023/7133726)

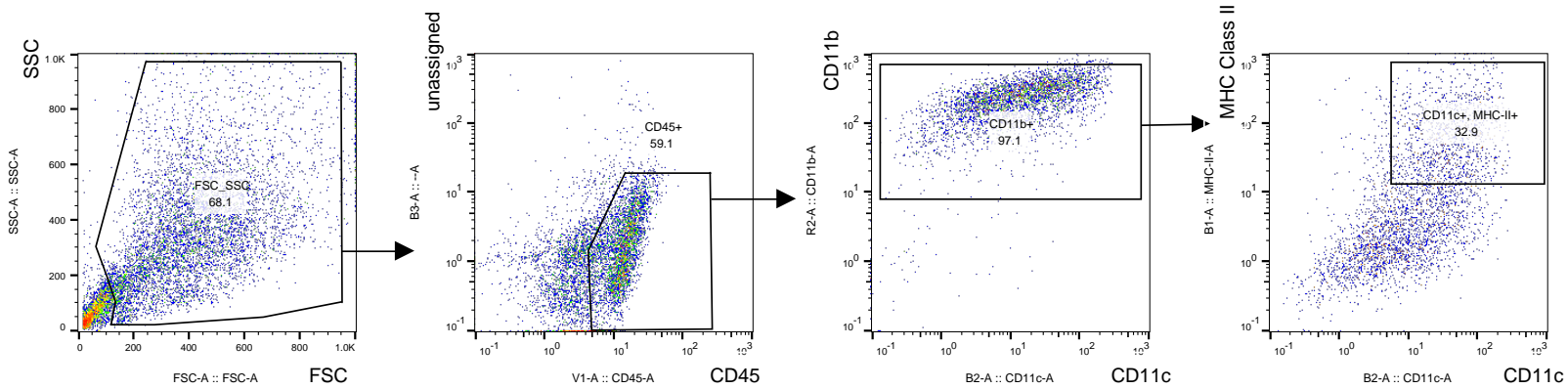

Supplement: Supplementary Materials — The supplementary information Figure S 1A-1B and Figure S 2A-2D are available in the supplementary material document. Figure S1: gating strategy for Figures 1(a) and 1(a). Figure S2: antitumor effect of ASP7657 in various tumors: (A–D) EMT-6 (1 × 10^5^ cells per mouse, A) and 4T1 (5 × 10^5^ cells per mouse, B) were subcutaneously inoculated into the right flank of BALB/c mice and LL/2(LLC1) (3 × 10^5^ cells per mouse, C) and B16-F10 (3 × 10^5^ cells per mouse, D) into the right flank of C57BL/6J mice. After tumors were established, mice were treated with ASP7657 twice per day at the indicated doses. Data show mean ± SEM (n = 9 − 10 per group). ∗p < 0.05, ∗∗p < 0.01, compared with the vehicle-treated group (Dunnett's multiple comparison test, except for LL/2(LLC1) (Student's t-test)). [file 7133726.f1.zip › FigS1A_r.pdf]

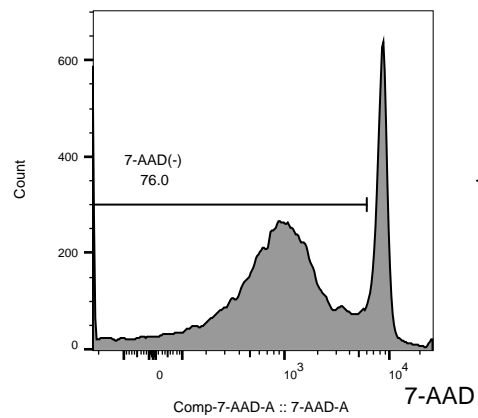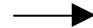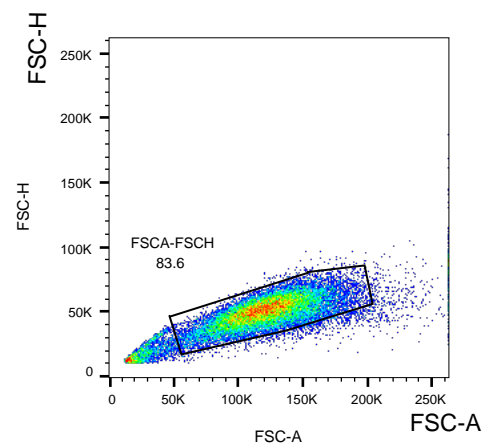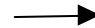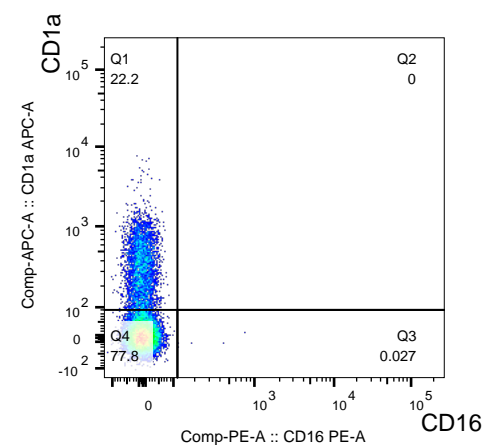

Supplement: Supplementary Materials — The supplementary information Figure S 1A-1B and Figure S 2A-2D are available in the supplementary material document. Figure S1: gating strategy for Figures 1(a) and 1(a). Figure S2: antitumor effect of ASP7657 in various tumors: (A–D) EMT-6 (1 × 10^5^ cells per mouse, A) and 4T1 (5 × 10^5^ cells per mouse, B) were subcutaneously inoculated into the right flank of BALB/c mice and LL/2(LLC1) (3 × 10^5^ cells per mouse, C) and B16-F10 (3 × 10^5^ cells per mouse, D) into the right flank of C57BL/6J mice. After tumors were established, mice were treated with ASP7657 twice per day at the indicated doses. Data show mean ± SEM (n = 9 − 10 per group). ∗p < 0.05, ∗∗p < 0.01, compared with the vehicle-treated group (Dunnett's multiple comparison test, except for LL/2(LLC1) (Student's t-test)). [file 7133726.f1.zip › FigS1B_r.pdf]

## EMT-6

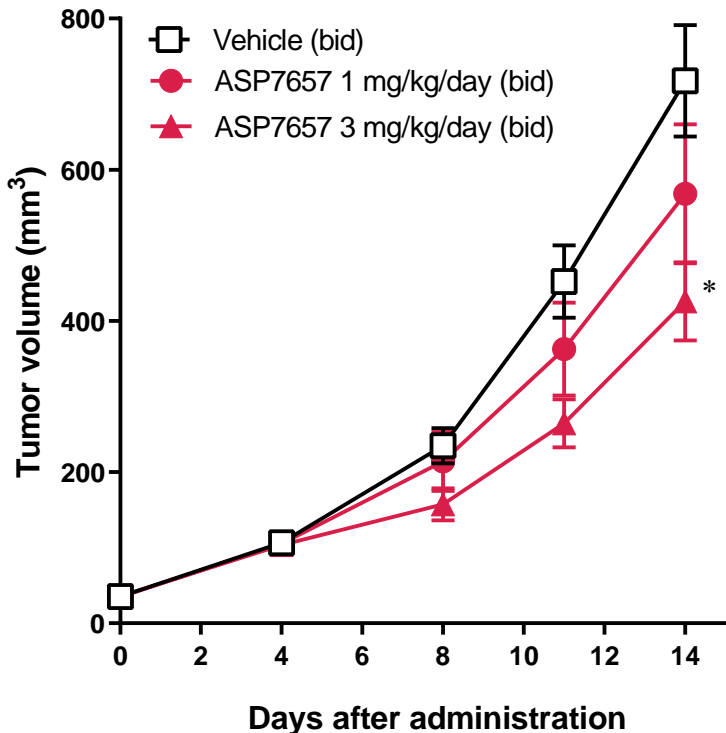

Supplement: Supplementary Materials — The supplementary information Figure S 1A-1B and Figure S 2A-2D are available in the supplementary material document. Figure S1: gating strategy for Figures 1(a) and 1(a). Figure S2: antitumor effect of ASP7657 in various tumors: (A–D) EMT-6 (1 × 10^5^ cells per mouse, A) and 4T1 (5 × 10^5^ cells per mouse, B) were subcutaneously inoculated into the right flank of BALB/c mice and LL/2(LLC1) (3 × 10^5^ cells per mouse, C) and B16-F10 (3 × 10^5^ cells per mouse, D) into the right flank of C57BL/6J mice. After tumors were established, mice were treated with ASP7657 twice per day at the indicated doses. Data show mean ± SEM (n = 9 − 10 per group). ∗p < 0.05, ∗∗p < 0.01, compared with the vehicle-treated group (Dunnett's multiple comparison test, except for LL/2(LLC1) (Student's t-test)). [file 7133726.f1.zip › FigS2A_r.pdf]

# 4T1

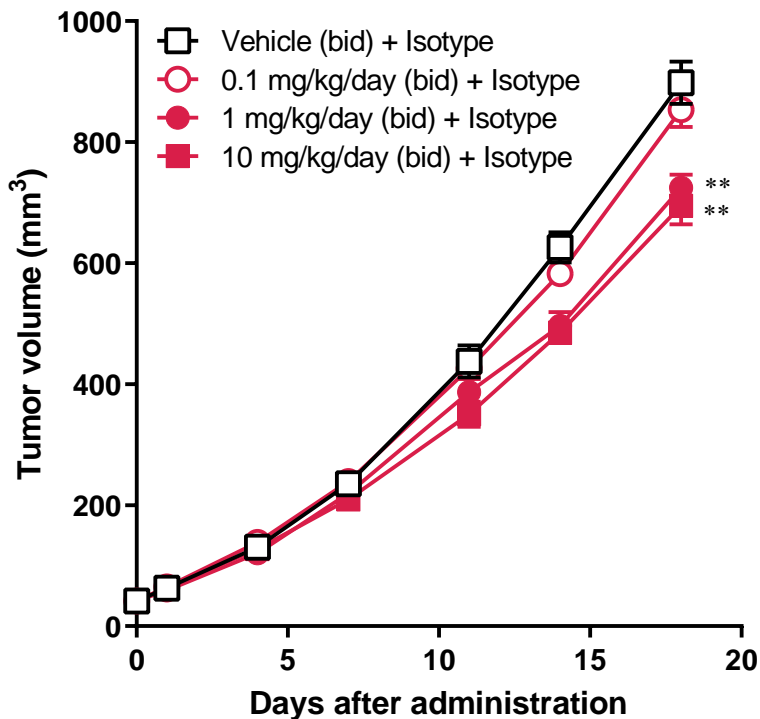

Supplement: Supplementary Materials — The supplementary information Figure S 1A-1B and Figure S 2A-2D are available in the supplementary material document. Figure S1: gating strategy for Figures 1(a) and 1(a). Figure S2: antitumor effect of ASP7657 in various tumors: (A–D) EMT-6 (1 × 10^5^ cells per mouse, A) and 4T1 (5 × 10^5^ cells per mouse, B) were subcutaneously inoculated into the right flank of BALB/c mice and LL/2(LLC1) (3 × 10^5^ cells per mouse, C) and B16-F10 (3 × 10^5^ cells per mouse, D) into the right flank of C57BL/6J mice. After tumors were established, mice were treated with ASP7657 twice per day at the indicated doses. Data show mean ± SEM (n = 9 − 10 per group). ∗p < 0.05, ∗∗p < 0.01, compared with the vehicle-treated group (Dunnett's multiple comparison test, except for LL/2(LLC1) (Student's t-test)). [file 7133726.f1.zip › FigS2B_r.pdf]

# LL/2(LLC1)

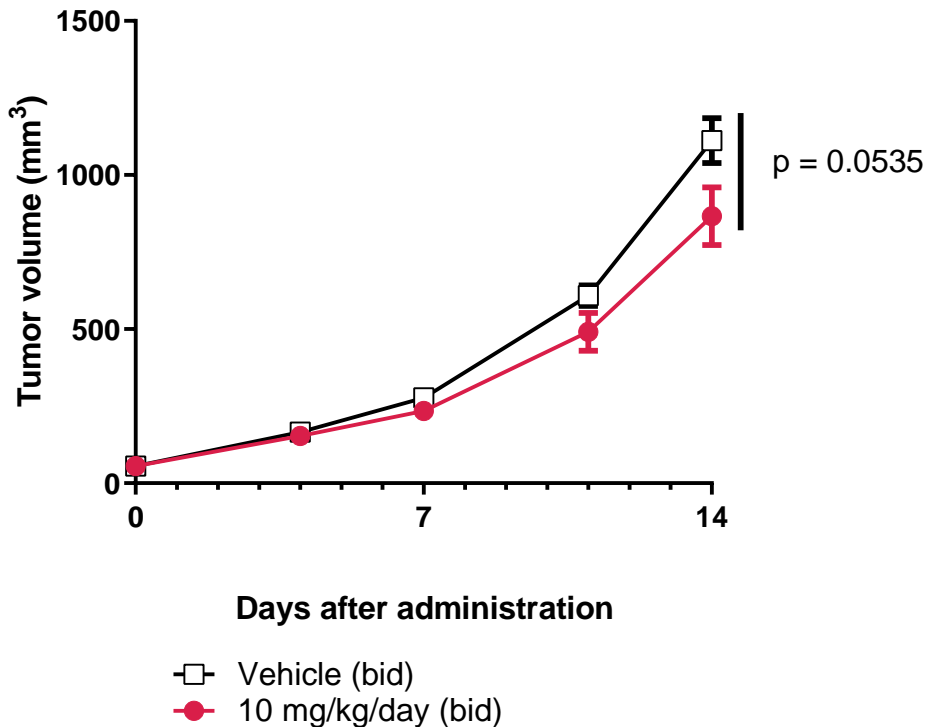

Supplement: Supplementary Materials — The supplementary information Figure S 1A-1B and Figure S 2A-2D are available in the supplementary material document. Figure S1: gating strategy for Figures 1(a) and 1(a). Figure S2: antitumor effect of ASP7657 in various tumors: (A–D) EMT-6 (1 × 10^5^ cells per mouse, A) and 4T1 (5 × 10^5^ cells per mouse, B) were subcutaneously inoculated into the right flank of BALB/c mice and LL/2(LLC1) (3 × 10^5^ cells per mouse, C) and B16-F10 (3 × 10^5^ cells per mouse, D) into the right flank of C57BL/6J mice. After tumors were established, mice were treated with ASP7657 twice per day at the indicated doses. Data show mean ± SEM (n = 9 − 10 per group). ∗p < 0.05, ∗∗p < 0.01, compared with the vehicle-treated group (Dunnett's multiple comparison test, except for LL/2(LLC1) (Student's t-test)). [file 7133726.f1.zip › FigS2C_r.pdf]

## B16-F10

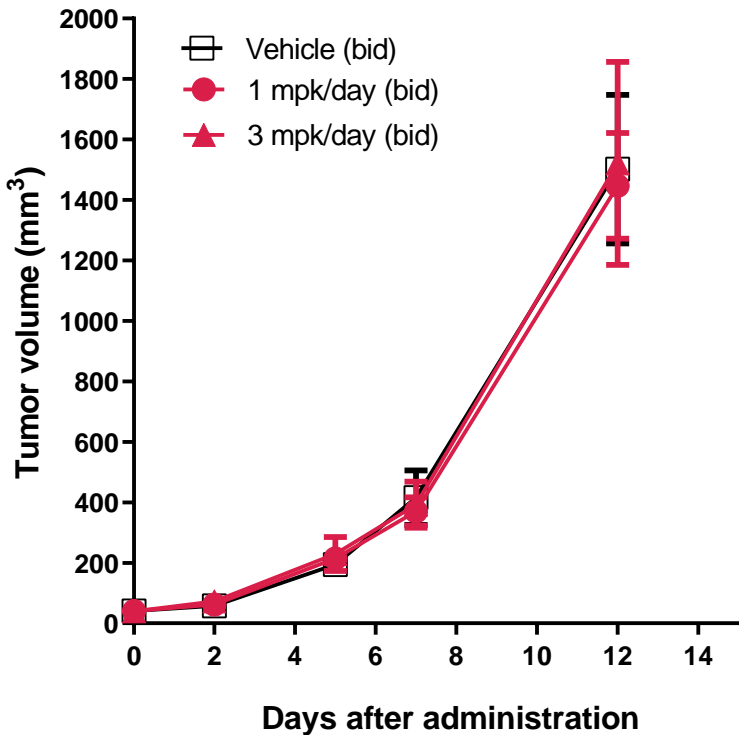

Supplement: Supplementary Materials — The supplementary information Figure S 1A-1B and Figure S 2A-2D are available in the supplementary material document. Figure S1: gating strategy for Figures 1(a) and 1(a). Figure S2: antitumor effect of ASP7657 in various tumors: (A–D) EMT-6 (1 × 10^5^ cells per mouse, A) and 4T1 (5 × 10^5^ cells per mouse, B) were subcutaneously inoculated into the right flank of BALB/c mice and LL/2(LLC1) (3 × 10^5^ cells per mouse, C) and B16-F10 (3 × 10^5^ cells per mouse, D) into the right flank of C57BL/6J mice. After tumors were established, mice were treated with ASP7657 twice per day at the indicated doses. Data show mean ± SEM (n = 9 − 10 per group). ∗p < 0.05, ∗∗p < 0.01, compared with the vehicle-treated group (Dunnett's multiple comparison test, except for LL/2(LLC1) (Student's t-test)). [file 7133726.f1.zip › FigS2D_r.pdf]
